# Exploring the Nursing Factors Related to Ventilator-Associated Pneumonia in the Intensive Care Unit

**DOI:** 10.3389/fpubh.2022.715566

**Published:** 2022-04-06

**Authors:** Yanling Yin, Meirong Sun, Zhe Li, Jingjing Bu, Yuhong Chen, Kun Zhang, Zhenjie Hu

**Affiliations:** Department of ICU, The Fourth Hospital of Hebei Medical University, Shijiazhuang, China

**Keywords:** ICU, VAP, nurse human resources, nursing practice, risk factors

## Abstract

**Objective:**

The purpose of this study was to investigate the key nursing factors associated with ventilator-associated pneumonia (VAP) in critical care patients.

**Methods:**

Through the quality control platform of Hebei Province, questionnaires were sent to intensive care nurses in 32 tertiary hospitals in Hebei Province, China to collect data concerning the incidence of VAP and the status of the nursing staff. All the data were analyzed using an independent *t*-test and a one-way analysis of variance (ANOVA). The Pearson correlation coefficient was used to analyse the correlation between the nursing factors and the incidence of VAP. Multivariate logistic regression analysis was used to determine the risk factors affecting VAP.

**Results:**

In terms of nursing, the incidence of VAP was affected by the differential nursing strategies. Multivariate logistic regression analysis showed that the incidence of VAP was significantly associated with the following six variables: the ratio of nurses to beds (*p* = 0.000), the ratio of nurses with a bachelor's degree or higher (*p* = 0.000), the ratio of specialist nurses (*p* = 0.000), the proportion of nurses with work experience of 5–10 years (*p* = 0.04), the number of patients nurses were responsible for at night (*p* = 0.01) and the frequency of oral care (*p* = 0.000).

**Conclusion:**

The incidence of VAP is closely related to nursing factors. In terms of nursing human resources, even junior nurses (less experienced nurses) can play an essential role in reducing VAP. In addition, to reduce VAP, the number of patients that nurses are responsible for at night should be reduced as much as possible, and improving nursing qualifications.

## Introduction

Mechanical ventilation is a commonly used rescue measure in hospital intensive care units (ICUs). Once a patient has respiratory failure, doctors will help the patient perform auxiliary breathing with the help of a ventilator (1), but complications, such as lung injury, pulmonary infections, ventilator-induced diaphragmatic dysfunction, and ventilator-associated pneumonia (VAP), often occur (2–5), the latter being the most common with severe cases causing damage to lung function. VAP refers to pneumonia developing in a mechanically ventilated patient more than 48 h after tracheal intubation (6, 7), and it is a common nosocomial infection. Studies have shown that VAP occurs in 10–40% of patients who are mechanically ventilated for more than 2 days (8, 9). The tracheobronchial colonization is one of the most critical factors for VAP, and *Staphylococcus aureus* and Enterobacteriaceae are the primary organisms that cause VAP infection (10).

Although some studies and guidelines refer to the use of cluster management to reduce the incidence of VAP (11), not all ICU nurses are the same because factors, such as educational levels, seniority, staffing, work shifts, and nursing levels, come into play (12). In recent years, many studies suggest reducing the incidence of VAP by adjusting the allocation of nursing human resources (13, 14). In addition, there are studies about the patient position, cuff pressure measurement, oral care, and analysis of the influence of effective coughing in patients with VAP (15).

The influence of nursing on VAP is mainly 2-fold: the nurses themselves and the nursing activities they undertake. However, previous studies have not systematically analyzed the aspect of nursing alone, and there has been a lack of detailed statistics to guide clinical nursing, leading to insufficient consideration of its potentially positive impact. Consequently, it is important to analyse the effect nursing has on the incidence of VAP to help reduce it and give patients a better prognosis.

## Methods

### Survey Respondents

The data used in this study were issued by the Hebei Provincial quality control platform. Questionnaire-based data were collected from 37 tertiary hospitals in Hebei Province, China. Five hospitals were excluded due to incomplete information, and finally, 32 hospitals were selected. The basic description of the hospitals is shown in Table 1.

**Table 1:** Information and demographic characteristics of the hospital.

**Items**		***n***	**%**
Level of hospital ^a^			
	3A	24	75
	3B	8	25
Total no. of beds		541	
Total no. of nurses		1,270	
Ratio of nurses to beds		2.35 : 1	
Working years of nurses in ICU
	≤ 5	485	38.18
	5–10	526	41.41
	>10	259	20.39
Highest degree of nurses
	Junior college degree or lower	313	24.64
	Bachelor degree or higher	957	75.35
Specialist nurse		188	14.8

a *According to the rule on hospital administration in China, hospitals are classified into 3 levels after review, and each level is divided into classes A, B, and C. Tertiary hospitals add special-grade, and the highest level of the hospital is tertiary special-grade*.

*3A, a tertiary class A hospital; 3B, a tertiary class B hospital*.

Inclusion criteria were as follows: 1) hospitals that participated in the questionnaire survey, collecting variables, included 21 entries regarding three areas on the acquisition platform of Hebei Province from January to December 2019. The first area was basic information, such as hospital type, the total number of beds, number of ICU beds, total number of nurses, nurse-to-bed ratio, and monthly incidence of VAP. The second area was nursing human resources that include the highest level of nursing education, the number of specialist nurses, and nurse shift arrangement. The third area related to nursing activities that include oral care tools, the method and frequency of oral care, the choice of oral care solution, the cuff pressure detection method and frequency of use, the size of the endotracheal tube, whether auscultation was performed before and after sputum aspiration, whether atomised inhalation was performed in mechanically ventilated patients, and whether the gastric residual volume was routinely monitored. 2) All the questionnaires were filled out by the ICU's head nurse, and the reported data were accurate and complete. This study was conducted in accordance with the Declaration of Helsinki and with approval from the Ethics Committee of the Fourth Hospital of Hebei Medical University. Written informed consent was obtained from all the participants.

### Survey Tools

After a literature review and research group analysis and discussion, a structured questionnaire was drawn up. Four hospital managers, nursing managers, and specialized nursing experts evaluated the clarity, rationality, comprehensiveness, and independence of the questionnaire items. The content validity index of the questionnaire was 0.89, indicating good content validity. The final questionnaire had two parts, Part 1 included the informed consent and instructions. Part 2 was the survey content that included questions about the hospital, the occurrence of VAP per month, the qualifications of the nurses, and related information on nursing activities (Table 1).

### Data Collection

The questionnaire was sent to the head nurse *via* e-mail to gather data about the quality of care and patient outcomes in each ICU ward. Finally, the questionnaires were consolidated by the investigator. A total of 32 questionnaires were sent out, and 32 were returned, making a recovery of 100%.

### Statistical Methods

All the data were statistically analyzed using SPSS 25.0 statistical software. Measurement data conforming to a normal distribution were presented as mean ± standard deviation (SD, $\bar{x}$ ± s). Categorical data were presented as absolute values with percentages. An independent sample *t*-test was used to compare data between two groups. A one-way analysis of variance (ANOVA) was used to compare data between multiple groups. Spearman's correlation analysis was used to analyse the correlation between different nursing factors and the incidence of VAP, and then multivariate logistic regression models were conducted to assess factors connected to VAP. *p* < 0.05 was considered statistically significant.

## Results

### Nursing Human Resources and Activities

There was a significant difference in the monthly VAP incidence amongst the 32 hospitals (*F* = 8.967, *p* < 0.001, see Supplementary Table S1). The classification conditions were based on nursing shift arrangements, nursing solution selection, oral care methods, balloon pressure monitoring methods, auscultation, whether the patient was aerosolised by device ventilation, and whether the gastric residue was routinely monitored. As shown in Table 2, there were significant statistical differences between the types of oral care methods (*p* = 0.007) and whether the patients with instrument ventilation received routine atomisation inhalation (*p* = 0.003) in the 32 included hospitals. There was no significant difference between nursing solution selection (*p* = 0.679), balloon pressure monitoring methods (*p* = 0.084), whether auscultation was conducted before sputum aspiration (*p* = 0.395), and whether gastric residue was routinely monitored (*p* = 0.819; Table 2).

**Table 2:** Nursing human resources and nursing operations.

**Factors**		**Total**	**Without**	**With**	***P***
		**number**	**VAP**	**VAP**	
Nurse shift schedule (%)	12 h	276 (71.9)	149 (72.3)	127 (71.3)	0.921
	8 h	108 (28.1)	57 (27.7)	51 (28.7)	
Oral care solution with antiseptic effect (%)	no	120 (31.2)	62 (30.1)	58 (32.6)	0.679
	yes	264 (68.8)	144 (69.9)	120 (67.4)	
oral_care_methods (%)	scrub	240 (62.5)	142 (68.9)	98 (55.1)	0.007
	rinse and scrub	144 (37.5)	64 (31.1)	80 (44.9)	
Monitoring method of cuff pressure (%)	use instruments	372 (96.9)	203 (98.5)	169 (94.9)	0.084
	use hands	12 (3.1)	3 (1.5)	9 (5.1)	
auscultation (%)	no	48 (12.5)	29 (14.1)	19 (10.7)	0.395
	yes	336 (87.5)	177 (85.9)	159 (89.3)	
use aerosol inhalations to treat mechanically ventilated patient (%)	no	168 (43.8)	105 (51.0)	63 (35.4)	0.003
	yes	216 (56.2)	101 (49.0)	115 (64.6)	
Gastric residual monitoring (%)	no	72 (18.8)	40 (19.4)	32 (18.0)	0.819
	yes	312 (81.2)	166 (80.6)	146 (82.0)	
oral_care_tools (%)	exclusive use package	312 (81.2)	168 (81.6)	144 (80.9)	0.69
	manual toothbrush	60 (15.6)	33 (16.0)	27 (15.2)	
	foam swabs	12 (3.1)	5 (2.4)	7 (3.9)	
suction tube sizes (diameter) (%)	10 Fr*	12 (3.1)	5 (2.4)	7 (3.9)	0.069
	12 Fr	252 (65.6)	140 (68.0)	112 (62.9)	
	14 Fr	60 (15.6)	37 (18.0)	23 (12.9)	
	16 Fr	60 (15.6)	24 (11.7)	36 (20.2)	

* *1 Fr = 0.33 mm*.

### The Correlation Between VAP and the Quality Factors of Nursing Care

The prevalence of VAP was taken as the dependent variable, and the proportion of nursing beds, education level, and time nursing was taken as the independent variables for correlation analysis. The results showed that the incidence of VAP was positively correlated with the proportion of nurses who had worked for <5 years (*p* < 0.01), the ratio of nurses to beds, the proportion of nurses with bachelor's degrees or higher and specialist nurses (*p* < 0.05), and the number of patients nurses were responsible for at night (*p* < 0.01). The incidence of VAP was significantly negatively correlated with the proportion of nurses who had worked for 5–10 years (*p* < 0.01) and the frequency of oral care (*p* < 0.01; Table 3).

**Table 3:** Correlation between ventilator-associated pneumonia (VAP) and related nursing factors.

	**Factors**	**Rate, number**,	***r***	***P***
	**of nursing**	**percentage**
Proportion of highest degree	Junior college degree or lower	25.79%	−0.096	0.06
	Bachelor degree or higher	72.73%	0.10	0.04
	Specialist nurse	0.72%	0.11	0.039
Years worked in ICU	≤ 5	37.26%	0.17	0.00
	5–10	43.28%	−0.19	0.00
	>10	19.06%	0.01	0.83
Nightshift frequency*		4.16 ± 0.61	0.04	0.42
Ratio of nurses to beds		2.44 ± 0.56	0.24	0.00
Number of patients managed by nurses	In day	2.28 ± 0.63	0.09	0.095
	In night	2.53 ± 0.71	0.17	0.00
Oral care frequency		3.06 ± 0.66	−0.18	0.00
Monitoring frequency of Cuff pressure		4.22 ± 1.48	0.02	0.69

* *Night shift frequency refers to a night shift occurring several days apart*.

### Oral Care Tools and Suction Tube Sizes

A one-way ANOVA was performed to compare the prevalence of VAP, taking the types of nursing tools and commonly used suction tubes as grouping conditions. There was a significant difference between the incidence of VAP and the choice of oral care tools (*p* = 0.007). There was no statistical difference with the selection of suction tube size (*p* = 0.069)(Table 2).

### Nursing Factors Affecting the Incidence of VAP

Multivariate logistic regression analysis showed that the incidence of VAP was significantly associated with the following six variables: the ratio of nurses to beds, the ratio of nurses with a bachelor's degree or higher, the ratio of specialist nurses, the proportion of nurses with work experience of 5–10 years, the number of patients nurses were responsible for at night, and the frequency of oral care (Table 4).

**Table 4:** Nursing factors affecting the incidence of ventilator-associated pneumonia (VAP).

**Risk factor**	**B**	**SE**	**OR**	***P***	***95%*** **CI**
Ratio of nurses to beds	1.217	0.27	3.38	0.00	(0.70, 1.93)
Ratio of bachelor degree or higher	0.023	0.01	1.02	0.00	(0.01, 0.04)
Ratio of specialist nurse	−0.38	0.14	0.69	0.00	(−0.75, −0.13)
Years worked in ICU					
≤ 5	0.01	0.01	1.01	0.56	(−0.01, 0.03)
5–10	−0.02	0.01	0.98	0.04	(−0.04, 0.00)
Number of patients managed by nurses in night	0.47	0.19	1.60	0.01	(0.10, 0.95)
Oral care frequency	−0.56	0.19	0.57	0.00	(−1.02, −0.14)

*B value refers to the regression coefficient and intercepts (constant term); SE is the standard error; OR is the odds ratio*.

## Discussion

Ventilator-associated pneumonia is a preventable iatrogenic disease that occurs after mechanical ventilation. Clustering is currently recommended to prevent VAP. Recent studies have also confirmed the effectiveness of the VAP prophylaxis package (16, 17). It can be seen that most of these measures were related to nursing (18). This study has found that nurse staffing and nursing practices affect the incidence of VAP (Table 4) in that the incidence of VAP had a positive relationship with the proportion of nurses who had worked for fewer than 5 years, which is discussed below, and the number of patients that nurses care for on the night shift.

A low number of nurses is associated with an increased risk of infection, and by keeping the number of staff at a high level, a significant proportion of all infections acquired in intensive care can be prevented (19). Dimick et al. investigated whether the ratio of nurses to patients at night had an impact on patients who suffered from complications after high-risk surgical procedures (liver surgery). They found that patients suffered from postoperative pulmonary complications when one nurse cared for three or more patients on the night shift in the ICU (20). Another study confirmed that a low nurse-to-patient ratio (<1:2) was associated with increased incidences of complications, such as pneumonia, re-intubation and sepsis, prolonged hospitalization, and increased costs (21). This is consistent with the findings of this study. However, one study found no association between high staffing levels (patient-to-nurse ratio <2:1) and reduced VAP risk, based on a large group of patients receiving mechanical ventilation from 21 European ICUs. Although a patient-to-nurse ratio of 1:1 in univariate analysis was associated with a lower risk of VAP, this observation was no longer important after adjusting for covariates (22). It is important to note that the present study only focused on the relationships between nursing factors and VAP.

Although many studies have shown no statistically significant difference between years of nursing service and the prevention of VAP (23–25), these studies all underscore the point that nursing training directly affects the incidence of VAP. In addition, the proportion of nurses with a bachelor's degree or above was significantly correlated with the incidence of VAP. Kutney-Lee et al. (26) analyzed the nurse survey and patient discharge data from 1999 to 2006 in Pennsylvania, USA. Their results showed that a 10% increase in the number of nurses with a bachelor's degree or higher reduced the average number of deaths of patients by 2.12 per 1,000 cases. Subgroup analysis showed that for patients with complications, a 10% increase in the number of undergraduate nurses reduced the number of deaths by 7.47 cases per 1,000 cases. Aiken et al. (27) found that a 10% increase in the number of nurses with a bachelor's degree and above led to a 7% decrease in the mortality rate of hospitalized patients. The American National Institute of Medicine recommends that hospitals give priority to undergraduate nurses, and by 2020, the proportion of undergraduate nurses in the United States was expected to have reached 80% (28). In short, highly educated nurses are required in the healthcare industry, as evidenced by the data suggesting that good nursing education tends to be associated with a lower incidence of VAP.

Ventilator-associated pneumonia is a common cause of death and morbidity in patients with endotracheal intubation, partly due to the low ratio of nurses to beds. A study in Jordan showed that in the prevention of VAP, the low ratio of nurses to beds could affect the compliance of nurses and the patient outcome (29). Another study showed that a low nurse-to-patient ratio could lead to an increase in the incidence of late-onset VAP (30). A sufficient number of nurses means that patients receive more care, and nursing problems can be discovered in time. However, there is no relevant research to confirm that a suitable nursing bed can prevent VAP without causing waste of personnel.

Nursing tools and medication are known to be amongst the causes of serious infection, such as VAP, in critically ill patients, in this case by damaging the oral mucosa of the patients (31–33). There is also evidence that good oral care can effectively prevent ICU-acquired infections, mainly VAP, and speed up recovery and discharge (34, 35), and the preservation or restoration of the oral mucosa depends on the frequency of oral care (36). Özlem and Banu (37) produced an oral care frequency assessment scale to better protect the oral mucosa of critically ill patients. The need for such assessment is consistent with the results of this study.

In conclusion, nursing can affect the incidence of VAP, the ratio of nurses to beds, the ratio of nurses with a bachelor's degree or higher, the ratio of specialist nurses, the proportion of nurses with work experience of 5–10 years, the number of patients nurses were responsible for at night, and the frequency of oral care was significantly associated with the incidence of VAP.

### Relevance to Clinical Practice

Nurses play an essential role in reducing the occurrence of VAP in intubated patients, the ratio of nurses to beds, the ratio of nurses with a bachelor's degree or higher, the ratio of specialist nurses, the proportion of nurses with work experience of 5–10 years, and the number of patients nurses were responsible for at night, and the frequency of oral care was significantly associated with the incidence of VAP. In addition, nursing managers need to optimize staffing. Through training and the integration of nursing human resources, nurses can better participate in their work, resulting in a reduction in the incidence of VAP.

## Data Availability Statement

The original contributions presented in the study are included in the article/Supplementary Material, further inquiries can be directed to the corresponding author.

## Ethics Statement

The studies involving human participants were reviewed and approved by Fourth Hospital of Hebei Medical University. The patients/participants provided their written informed consent to participate in this study.

## Author Contributions

YY, MS, and YC conceived the study. ZL and KZ participated in its design and coordination. ZH and JB helped draft the manuscript. All authors read and approved the final manuscript.

## Funding

Study on treatment status and prognostic risk factors of severe COVID-19 patients in Hebei Province, Project No. 20277707D.

## Conflict of Interest

The authors declare that the research was conducted in the absence of any commercial or financial relationships that could be construed as a potential conflict of interest.

## Publisher's Note

All claims expressed in this article are solely those of the authors and do not necessarily represent those of their affiliated organizations, or those of the publisher, the editors and the reviewers. Any product that may be evaluated in this article, or claim that may be made by its manufacturer, is not guaranteed or endorsed by the publisher.
