# Supplementary material for: Exploring the Nursing Factors Related to Ventilator-Associated Pneumonia in the Intensive Care Unit
Source: Front Public Health. 2022 Apr 6;10:715566. doi: 10.3389/fpubh.2022.715566 (PMC9019058; doi:10.3389/fpubh.2022.715566)
Supplement: Supplementary file 1 [file Table_1.pdf]

Hospital	the occurrence of VAP per month (%)											
	Jan	Feb	Mar	Apr	May	Jun	Jul	Aug	Sep	Oct	Nov	Dec
1	0	0	0	0	0	0	0	0	0	0	0	0
2	0	1.67	3.24	1.78	0	1.86	3.99	13.54	1.57	1.37	0	4.44
3	35.7	35.6	37.8	43.2	43.5	38	23	20.6	19.6	19.9	29.1	23.3
4	6.66	15.1	0	0	21.5	0	0	0	0	17.2	0	0
5	4.44	6.29	0	12.58	0	14.29	25.64	7.52	0	10.49	12.88	4.42
6	0	0	0	0	4.76	0	0	0	0	0	6.8	0
7	0	0	0	0	0	0	0	0	0	0	0	0
8	2.6	0	2.4	9.9	10.6	0	9.6	0	17.6	9.8	6.6	13.3
9	0	0	0	0	11.49	0	0	0	0	0	0	0
10	23.26	17.86	0	0	20.83	18.18	0	19.23	0	16.67	18.52	64.52
11	0	0	2.76	0	0	0	0	0	2.81	0	0	0
12	13.82	0	0	5.99	19.42	7.35	0	0	24.1	3.02	10.63	0
13	18.78	24.29	6.33	10.58	0	6.9	30.61	21.98	17.02	14.98	11.72	21.01
14	0	0	9.71	0	0	0	0	0	14.49	0	0	0
15	0	0	0	0	0	0	16.67	0	0	0	0	0
16	0	0	6.45	5.71	0	16	7.25	9.26	11.49	0	6.62	0
17	0	0	0	0	0	9.43	0	3.05	6.1	0	2.71	5.42
18	0	0	0	0	0	0	5.68	0	0	6.6	3.8	3.07
19	0	9.62	0	0	8.93	0	0	0	0	0	0	0
20	7.12	3.02	2	0	7.95	0	6.32	9.22	11.21	5.32	8.23	5.31
21	0	0	0.7	0	2.4	2.38	0	0	0	0	0	12.66
22	3.02	0	0	0	0	0	0	7.14	0	11.63	13.57	13.1
23	0	0	3.2	0	2.5	0	6.9	3.3	2.3	2.8	7.5	0
24	0	0	0	7.76	0	0	0	18.87	0	0	0	0

25	0	6.15	0	2.3	1.8	0	0	3.7	4.5	2.7	0	0
26	18.52	0	4.26	0	10.87	6.58	0	24.39	5.92	0	15.63	15.23
27	2.77	0	3.36	0	3.33	0	3.81	0	3.98	2.61	3.91	3.48
28	0	17.86	0	0	0	0	0	0	0	38.46	61.73	54.05
29	0	0	1	0	0	0	0	7.09	28.85	0	3.91	6.62
30	0	10.05	6.17	4.57	0	0	0	0	0	12.74	0	9.22
31	4.88	0	18.18	12.5	5.68	12.99	0	8.4	0	0	10.1	0
32	5.05	6.89	4.18	7.14	4.94	7.83	5.43	5.74	6.64	7.54	5.35	3.66

Supplementary Table 1
